# Correction: Phase-amplitude coupling supports phase coding in human ECoG

**DOI:** 10.7554/eLife.12810

**Published:** 2015-11-06

**Authors:** Andrew J Watrous, Lorena Deuker, Juergen Fell, Nikolai Axmacher

Watrous AJ, Deuker L, Fell J, Axmacher N. 2015. Phase-amplitude coupling supports phase coding in human ECoG. *eLife* **4**:e07886. doi: 10.7554/eLife.07886

Published 26 August 2015

In the published article, “χ^2^” was incorrectly rendered as “|2” in the following sentences:

“Slow-modulating (‘Fphase’) frequencies were significantly clustered in the delta band (0.5–4 Hz; Figure 2G) and HFA modulated frequencies (‘Famp’) were significantly clustered around slow (∼32 Hz) and fast (∼110 Hz) gamma frequencies (Figure 2H, chi-square goodness of fit test across gamma frequencies, p < 0.004, χ^2^(22) = 43.6, Cohen's d = 0.77).”

“In fact, across electrodes, phase-coding was equally likely to occur at all phases and for all categories; phase-coded categories were not clustered at particular phases at any level of DS (Rayleigh test, all p > 0.19; Figure 3F) and phase-coding was equally likely for each category (χ^2^(3) = 1.6, p = 0.64).”

Additionally, in the caption for Figure 3—figure supplement 3, “exclusively for houses” should have read “exclusively for houses, tools, scenes, or faces”.

In the funding section, the funder for grant SFB 1089 should have been “Deutsche Forschungsgemeinschaft” not “Australian Society for Fish Biology”.

The article has now been corrected.

